# Improvement in the Stability and Bioaccessibility of Carotenoid and Carotenoid Esters from a Papaya By-Product Using O/W Emulsions

**DOI:** 10.3390/foods12142654

**Published:** 2023-07-10

**Authors:** Sara Lara-Abia, Gloria Lobo, Noelia Pérez-Pascual, Jorge Welti-Chanes, M. Pilar Cano

**Affiliations:** 1 Laboratory of Phytochemistry and Plant Food Functionality, Biotechnology and Food Microbiology Department, Institute of Food Science Research (CIAL) (CSIC-UAM), Nicolas Cabrera 9, 28049 Madrid, Spain; sara.lara.abia@gmail.com (S.L.-A.); nellyper2000@gmail.com (N.P.-P.); 2 School of Sciences and Engineering, Tecnologico de Monterrey (ITESM), Monterrey 64849, NL, Mexico; jwelti@tec.mx; 3 Department of Crop Production in Tropical and Subtropical Areas, Instituto Canario de Investigaciones Agrarias (ICIA), 38297 Tenerife, Spain; globo@icia.es

**Keywords:** *Carica papaya* L., by-products, carotenoids, O/W emulsions, encapsulation, vegetable oils, stability, bioaccessibility, emulsion microstructure

## Abstract

The aim of the present work was to improve the stability and bioaccessibility of carotenoids from green oil extracts obtained from papaya by-products using oil-in-water (O/W) emulsions. The effects of different concentrations of pectin (1%, 2%, and 3%), a high-molecular-size emulsifier, together with Tween 20, a low-molecular-size emulsifier, high-speed homogenization conditions (time: 2, 3, 4, and 5 min; rpm: 9500, 12,000, 14,000, and 16,000 rpm), and high-pressure homogenization (HPH) (100 MPa for five cycles) were evaluated to determine the optimal conditions for obtaining O/W stable emulsions with encapsulated carotenoids. Soybean, sunflower, and coconut oils were used to formulate these O/W emulsions. The bioaccessibility of the main individual encapsulated papaya carotenoids was evaluated using the INFOGEST digestion methodology. In addition, the microstructures (confocal and optical microscopy) of the O/W carotenoid emulsions and their behavior during in vitro digestion phases were studied. Sunflower O/W carotenoid emulsions showed smaller mean particle size, higher negative ζ-potential, and higher viscosity than soybean O/W emulsions. Particle size reduction in the O/W emulsions using the HPH process improved the bioaccessibility of papaya encapsulated carotenoids. In these O/W emulsions, depending on the vegetable oil, lycopene was the carotenoid with the highest bioaccessibility (71–64%), followed by (*all*-*E*)-β-carotene (18%), (*all*-*E*)-β-cryptoxanthin (15%), and (*all*-*E*)-β-cryptoxanthin laurate (7–4%). These results highlight the potential of using green carotenoid papaya extracts to formulate O/W emulsions to enhance carotenoid bioactivity by efficiently preventing degradation and increasing in vitro bioaccessibility.

## 1. Introduction

In the last few years, a number of studies have proved that consuming carotenoids may provide health benefits, such as reducing the incidence of certain types of cancers, reducing the risk of cardiovascular disease, and improving eye health. Papaya fruits are rich sources of bioactive compounds such as carotenoids. Several studies have characterized their carotenoid profile, reporting that the most abundant carotenoids are β-carotene, β-cryptoxanthin and some of its isomers (such as laurate and caprate), and lycopene [1,2]. The Sweet Mary papaya variety is a hybrid cultivated in Tenerife, Canary Islands (Spain), and was recently introduced onto the market. Its physical–chemical properties (° Brix, color, shape, weight, etc.) and sensorial characteristics have made the Sweet Mary papaya popular among consumers. Papaya pulp is the edible part of the plant, and numerous studies have used it as a starting material due to the interest in its high content of bioactive compounds. However, papaya peel is considerably less studied than pulp and is usually discarded during the production process, generating waste and by-products. However, according to published studies, papaya peel is also a rich source of bioactive compounds, such as carotenoids [3]. Carotenoids are lipophilic compounds susceptible to oxidation and degradation, especially once extracted from their original tissues [4,5]. To exert their function in the human body, they must be released from the food matrix and become available for absorption. However, most of them present low availability (5–30%) compared to other food phytochemicals [6]. A previous study reported by our group [7] showed low bioaccessibility of the individual carotenoids from papaya pulp (Sweet Mary variety) exposed to the high hydrostatic pressure (HHP) process. In that work, the bioaccessibility values reported for β-cryptoxanthin, β-cryptoxanthin laurate, β-carotene, and lycopene ranged from 0 to 3% in the control and HHP-treated samples, respectively, indicating that there was no improvement in carotenoid bioaccessibility due to this process. Interest in incorporating carotenoids into functional food products has grown over the last few years. To design a functional ingredient with enhanced bioavailability of carotenoids, the formulation of O/W emulsions (micro- and nanoemulsions) or capsules for addition to foods could be an excellent strategy with promising results. A recent review on the application of advanced emulsion technology in the food industry [8] reported that advanced emulsions feature superior properties over conventional emulsions for delivering bioactive compounds, associated with higher retention efficiency, increased stability against environmental stresses, improved bioavailability, and the ability to control or trigger release. In the case of carotenoids, Xavier et al. [9] obtained a lutein bioaccessibility of up to 48% after incorporating a lutein emulsion into a yogurt, where the bioaccessibility value depended on the fat content of the yogurt. García-Cayuela et al. [10] reported that persimmon provitamin A carotenoids showed improved bioaccessibility by up to 38% when lyophilized fruit pulp was incorporated into whole cow’s milk (3.6% fat). In general, emulsion-based delivery systems such as oil-in-water (O/W) emulsions are widely used to protect lipophilic bioactive compounds, i.e., carotenoids, from degradation and oxidation, enhancing their dispersion in aqueous media and increasing their bioaccessibility and stability under gastrointestinal conditions. During digestion, lipids are mandatory for the micellarization of carotenoids since they favor the dissolution of carotenoids into fat droplets in the gastric emulsion environment. Furthermore, the intake of fats may delay gastric emptying, with the fats forming more micelles. Moreover, the nature of triacylglycerol molecules (fatty acid chains and saturation degree) also influences the bioaccessibility of carotenoids [10,11]. Other studies showed that carotenes present higher bioaccessibility when they are consumed together with foods containing long-chain triacylglycerides, while xanthophylls need medium-chain triacylglycerides [12]. The presence of pectin also influences carotenoid bioaccessibility in different ways. In emulsions, pectin could help to reduce the interfacial tension between the lipid and the aqueous phases, increasing the viscosity of the aqueous phase. In food emulsions, pectin is used as an emulsifier and stabilizing agent [13], and some studies have reported the impact of food pectin on health, such as the satiety effect, prevention of some gastrointestinal diseases, and glycemia control [14]. Therefore, systems based on the formulation of carotenoid emulsions with pectin as an emulsifier could be studied under gastrointestinal conditions to evaluate their efficiency in improving their individual stability and bioaccessibility. Teixé-Roig et al. [15] reported improved bioaccessibility of β-carotene (standard) by up to ≈36% using nanoemulsions formulated with pectin. Other formulations have been used to encapsulate carotenoids. For example, in their study, Jain et al. [16] described the effect of modified rice-starch-based delivery systems on lycopene storage stability and bioaccessibility. In their work, lycopene showed a significantly higher bioaccessibility when it was encapsulated with the emulsions (20.2%) compared to the alginate beads (15.6%), whereas the chemical stability of lycopene was found to be significantly higher in the alginate beads (35.6%) than in the emulsions (29.5%). In the literature, some studies have reported the use of oil-in-water nanoemulsions with lactoferrin and different carrier oils to improve lycopene stability and bioaccessibility [17], but there are few published studies on the encapsulation of all carotenoids present in direct extracts (non-saponified) from papaya by-products with O/W emulsions. These studies would provide information about the stability and bioaccessibility of each individual carotenoid, including the xanthophyll esters, during in vitro gastrointestinal digestion.

In the present study, soybean oil and sunflower oil (rich in long-chain fatty acids) and coconut oil (TCM) (rich in medium-chain fatty acids) enriched with direct carotenoid extracts, obtained from a Sweet Mary papaya by-product (peels), were used to formulate oil-in-water (O/W) emulsions. The physical characteristics (particle size, zeta potential, and viscosity) and microstructure of the emulsions were studied. The digestive stability and bioaccessibility of the main individual O/W-encapsulated papaya carotenoids (including xanthophyll esters) were determined using the standardized in vitro INFOGEST gastrointestinal simulation method. In addition, the stability of the emulsion structures during in vitro digestion was studied using optical microscopy. This study could provide more information about the potential use of formulated papaya by-product extracts to obtain a healthy ingredient.

## 2. Materials and Methods

### 2.1. Chemicals, Standards, and Reagents

Ultrapure water was obtained from a Millipark^®^ Express 40 system (Merk- Millipore, Darmstadt, Germany); methanol, diethyl ether, tetrahydrofuran (THF), methyl tert-butyl ether (MTBE), acetone, and petroleum ether were purchased from VWR International (Radnor, PA, USA). Butylated hydroxytoluene (BHT) and magnesium carbonate were obtained from Acros Organics (New Jersey, NY, USA). Anhydrous sodium sulfate, sodium chloride (NaCl), and potassium hydroxide (KOH) were purchased from Panreac Quimica (Barcelona, Spain). Standards for lycopene (L9879, ≥90%, from tomato), (*all*-E)-β-apo-8′-carotenal (10,810, ≥96%, (UV)), and lutein (X6250 from marigold) were purchased from Sigma-Aldrich (St. Louis, MO, USA). Standards for (*all*-E)-β-carotene (HPLC 96%, synth., cryst.), (*all*-E)-α-carotene (HPLC 97%, synth., cryst.) and (*all*-E)-β-cryptoxanthin (HPLC 97%, synth., cryst.), (*all*-E)-violaxanthin (HPLC 95%, isolated, cryst.), (*all*-E)-neoxanthin (HPLC 97%, isolated, cryst.), and (*all*-E)-zeaxanthin (HPLC 97%, synth., cryst.) were purchased from CaroteNature (Ostermundigen, Switzerland). Enzymes used in the simulated gastrointestinal in vitro digestion were acquired from Sigma-Aldrich (St. Louis, MO, USA). The following enzymes were used: α-amylase from porcine pancreas (10,080; 79 U mg^−1^), pepsin from porcine gastric mucosa (P6887; 791 U mg^−1^), pancreatin from porcine pancreas (P7545; 17 units TAME per mg), and bile salts from bovine and ovine origin (B8381). Other reagents used in the in vitro digestion assay were acquired from Sigma-Aldrich (St. Louis, MO, USA). Tween 20 was obtained from Sigma-Aldrich (St. Louis, MO, USA). Food-grade high-methoxyl pectin from citrus peel with 67% to 71% methylesterification was obtained from Acros Organics (Morris Plains, NJ, USA). Coconut oil (MCT, medium-chain triglyceride) was purchased from Shanghai YuanYe Biological Technology Co., Ltd., (Shanghai, China) and soybean (La Española Soy Plus^®^) and sunflower (Aliada^®^) refined oils were purchased from a local market in Madrid (Spain). The fatty acid composition of vegetable oils from Dubois et al. [17] is presented in Table S1 (Supplementary Materials).

### 2.2. Papaya By-Product

The Sweet Mary papaya (*Carica papaya* L.) fruit by-product used in the study was obtained from Tenerife (Canary Islands, Spain: 28°18′52′′ north (N); 16°24′36′′ west (W); 271 m above sea level). Papaya fruits that were not suitable for commercialization (not uniform in size) were washed and selected according to peel coloration and ripeness. After they were washed and drained, they were cut, the seeds were removed, and pulp and peel (<2 mm) tissues were separated. Uniform sections (1 cm^3^) of papaya peel tissue were vacuum-packed in 200 mm × 300 mm plastic bags (Cryovac^®^, Sealed Air Corporation, Madrid, Spain), frozen with liquid nitrogen, and freeze-dried for 5 days at −45 °C and 1.3 × 10^−3^ MPa (LyoBeta 15, Azbil Telstar SL, Terrasa, Spain). The freeze-dried material was ground by pulverizing (Grindomix GM200, Retsch, Haan, Germany) to a fine particle size (<2 mm), vacuum-packed in plastic bags, and stored at −80 °C until carotenoid analysis. For the present study, freeze-dried papaya peel was selected as a model papaya by-product to obtain direct carotenoid extracts to formulate O/W emulsions.

### 2.3. Carotenoid Extraction from Freeze-Dried Papaya Peel

Carotenoids and carotenoid esters were extracted according to the method of Cano et al. [18], with some modifications. The extraction was carried out under dim light, using amber flasks and avoiding long-term oxygen exposure. First, 1 g of freeze-dried sample was mixed with 0.5 g of magnesium carbonate. Then, 20 mL of tetrahydrofuran (THF) stabilized with 0.01% (*w*/*v*) butylated hydroxytoluene (BHT) was added for the extraction. The sample was homogenized in an Omnimixer (OMNI Macro S^®^, OMNI International, Kennesaw, GA, USA) for 3 min at 7000 rpm and placed in an ultrasonic water bath (3000514 model, 50/60 Hz, 360 W, J. P. Selecta S.A., Barcelona, Spain) for 30 min. The extract was centrifuged at 9000 rpm for 10 min at 4 °C, and the supernatant was collected. Next, 20 mL of acetone was added to the pellet and the sample was extracted again. This step was repeated until a colorless pellet was obtained. The supernatants were combined and placed in a separation funnel, adding 20 mL of diethyl ether. The funnel was shaken, and the organic phase was collected in a round amber flask. If an emulsion was formed, 10 mL of ultrapure water was added. This step was repeated twice. The organic phase was collected and dried with 2.5 g of anhydrous sodium sulfate for 10 min at room temperature. Then, the extract was filtered through Whatman No. 1 filter paper and vacuum-dried under a steam of nitrogen. Finally, the extract was kept at −80 °C until use in emulsion preparations and for HPLC analysis.

### 2.4. Preparation of Papaya By-Product Carotenoid O/W Microemulsions

The microemulsions were prepared using a reported method [15] with modifications. Elaboration of the O/W emulsions without carotenoid extracts was carried out in order to assess the best conditions to obtain stable emulsions (results not included) to prepare the O/W carotenoid-enriched emulsions.

Briefly, an oil-in-water emulsion containing papaya carotenoid extract was prepared in the dark to prevent the degradation of carotenoid by light. The oil phase was prepared by completely dissolving 7 mg of carotenoid extract in 1 g of vegetable oil (soybean, sunflower, or coconut oils). The aqueous phase consisted of pectin (1%, 2%, and 3%) dissolved in ultrapure water preheated at 70 °C 1% (*w/w*) and homogenized using an Ultraturrax homogenizer (T-25 Digital, IKA work Inc., Breisgau, Germany) at 9500 rpm for 5 min. Then, this aqueous phase was left for 1 h to cool at room temperature.

To obtain the coarse emulsion, in the first stage, the lipid phase (4% *w/w*), Tween 20 (4% *w*/*w*) as a low-molecular-weight emulsifier, and the aqueous phase containing pectin were homogenized at different speeds (9500, 12,000, 14,000, and 16,000 rpm) with 1 s ON/1 s OFF pulses and continuous mode for each speed, using an Ultraturrax homogenizer (T-25 Digital, IKA work Inc., Breisgau, Germany). In the second stage, a high-pressure homogenization process using an advance Panda PLUS 2000 GEA Niro Soavi (Parma, Italy) was employed. This device is composed of two valves. In valve no. 1, the pressure was set at 90 MPa to micronize the sample in stage 1. In valve no. 2, the operational pressure was set at 100 MPa to disperse the sample in stage 2, obtaining stable emulsions. After setting the parameters, the coarse emulsion was passed through the high-pressure homogenizer 5 times (5 cycles) under these conditions.

### 2.5. Characterization of O/W Microemulsions

#### 2.5.1. Particle Size and Zeta Potential

The particle size of emulsions was measured using a Mastersizer 3000 (Malvern Instruments Ltd., Worcestershire, UK). Samples were diluted in ultrapure water and stirred in the dispersion unit at a constant speed of 2000 rpm. The particle size was expressed as average diameter volume-to-surface (d32) in micrometers (μm), using the refractive index 1.333 for water. The electrical charge (ζ-potential) was measured through phase-analysis light scattering (PALS) using a Zetasizer NanoZS (Malvern Instruments Ltd., Worcestershire, UK) to determine the surface charge at the interface of the droplets. Emulsions were diluted (1:10) in ultrapure water and placed in a capillary cell equipped with two electrodes to assess the electrophoretic mobility of the particles. The results are reported in millivolts (mV).

#### 2.5.2. Microstructure of O/W Carotenoid Microemulsions and In Vitro Gastrointestinal Digestion Phases

Images of the coarse emulsion O/W, the O/W carotenoid microemulsions, and the corresponding O/W microemulsions during in vitro gastrointestinal digestion were obtained using an optical microscope Axioskop (Carl Zeiss, Germany) coupled to a Leica DMC 6200 pixel shift camera (Leica Microsystems, Germany), using a Zeiss Plan-Neofluar lens with a 100× objective and the addition of an immersion oil drop. Samples were observed using an open condenser with level 4 illumination, and no color filters were used. The color was manually adjusted to show real-time colors using Leica LAS v4.13 Application Suite Software.

Microstructural analysis was also performed using confocal laser scanning microscopy (CLSM). Neutral red solution (50 μg/mL in ethanol, Sigma, Deisenhofen, Germany) was used to dye the lipophilic phase of the emulsions. The excitation and emission peaks of the dye were at 515 nm and 585 nm, respectively. This analysis was achieved using a confocal multispectral TCS SP8 system (Leica Microsystems, Mannheim, Germany) at 40× with a Zeiss Plan-Neofluar lens.

### 2.6. Viscosity

The viscosity of O/W emulsions was measured using a Haake high-temperature viscometer (IKA^®^—Werke GmbH & Co., KG, Staufen, Germany) with an ME 1700 sensor system. The methods used are described in the ISO 7884-2 and ISO 7884-3 standards (www.iso.org, 2020).

### 2.7. Measurement of Encapsulation Rate

The encapsulation efficiency (EE) (%) was calculated as (the total carotenoids in the emulsion/total carotenoids in the extract used to formulate the emulsion) × 100. The total carotenoid content was determined both through the spectrophotometry method using β-carotene as the standard and though the sum of individual quantified carotenoids analyzed using HPLC. The extraction protocol to analyze the carotenoids encapsulated by the O/W emulsions is described in Section 2.8.

### 2.8. Carotenoid Extraction from O/W Microemulsions

The procedure reported by Teixé-Roig et al. [15] with some modifications was followed to extract the carotenoids from the O/W microemulsions. First, 5 mL of the O/W microemulsion was mixed with 5 mL of chloroform, and the mixture was centrifuged at 3000 rpm for 20 min at 4 °C. After centrifugation, the chloroform phase (orange-colored) was collected. This process was repeated 3 times. Then, the extract was dried in a rotatory evaporator at 20 °C, re-suspended in 2 mL of MeOH:MTBE:H_2_O (45.5:52.5:2, *v/v/v*), filtered through a 0.45 μm filter, and analyzed through HPLC and by spectrophotometry for total carotenoid content.

### 2.9. Characterization and Quantification of Carotenoids from Papaya Peel (By-Product)

#### 2.9.1. Analysis of Carotenoids using HPLC

The carotenoids (hydrocarbon carotenoids, free xanthophylls, and xanthophyll esters) were quantified simultaneously through high-performance liquid chromatography using a 1200 Series Agilent HPLC System (Agilent Technologies, Santa Clara, CA, USA) with a reverse-phase C30 column (YMC-Pack YMC C30, 250 × 4.6 mm inner diameter (i.d.), S-5 μm, YMC Co., Ltd., Kyoto, Japan) at 32 °C. The mobile phases used for the separation were MeOH:MTBE:H_2_O (81:14:4, *v/v/v*, eluent A) and MeOH:MTBE (10:90, *v/v*, eluent B), both containing 0.1% ammonium acetate [19]. The UV–vis photodiode array detector was set at 450 nm. UV–vis spectra were recorded between 200 and 700 nm. The LC–MS/MS (APCI^+^) analyses were performed using HPLC coupled to a mass spectrometry detector with an APCI source model G1947B (Agilent) compatible with LCMS SQ 6120 equipment [16]. Nitrogen was used as the drying gas at a flow rate of 60 L/min and as a nebulizing gas at a pressure of 50 psi. The nebulizer temperature was 350 °C, and a potential of +2779 kV was used on the capillary. Helium was the collision gas, and the fragmentation amplitude was 0.8–1.2 V. The vaporizer temperature was set at 400 °C, and the corona was 4000 nA as a positive ion mode. The positive ion mass spectra of the column eluted at 13,000 Th/s (peak width 0.6 Th, FWHM).

Each carotenoid was identified according to its elution time, chromatography with carotenoid standards, the UV–visible spectrum (λ**_max_**, spectral fine structure (%III/II), peak cis intensity), and the mass spectrum as reported by Lara-Abia et al. [3]. The carotenoids were quantified using linear calibration curves prepared with concentrations of commercial standards in the range of 5–100 μg/mL of carotenoid stock solutions. Antheraxanthin and its isomers were quantified using the violaxanthin standard curve. Figure S1 (Supplementary Materials) shows the C30 HPLC reversed-phase chromatogram of the papaya peel carotenoids, and Table S2 displays all identified carotenoids present in the vegetable–papaya–carotenoid-enriched oils.

#### 2.9.2. Analysis of Total Carotenoids through Spectrophotometry

Total carotenoids were analyzed using spectrophotometry at 420 nm. For this, the dried extract obtained from papaya peel was dissolved in hexane, β-carotene was used as the standard, and the β-carotene extinction coefficient was used in hexane 2560 [20]. This same papaya extract was used for the HPLC analysis of individual carotenoids, but in this case, the dried extract was dissolved in MeOH:MTBE:H_2_O (45.5:52.5:2, *v/v/v*), as noted in Section 2.8.

### 2.10. Bioaccessibility Study of Papaya Carotenoid Extracts Encapsulated by O/W Microemulsions

#### 2.10.1. In Vitro Gastrointestinal Digestion Assay

The in vitro gastrointestinal digestion assay was performed based on the standardized INFOGEST© protocol [21] with the adaptations for carotenoid studies reported by Cano et al. [19]. Microemulsions were digested immediately after their preparation.

Digestive solutions for the mouth (simulated saliva fluid, SSF), stomach (simulated gastric fluid, SGF), and small intestinal (simulated duodenal fluid, SDF) compartments were prepared following the methodology described by Eriksen et al. [22]. To avoid the loss of activity and denaturalization, enzyme solutions were prepared daily prior to the digestive assay. After each phase (oral, gastric, and intestinal) of the in vitro digestion, a digestive sample was obtained, named digesta. The digesta was ultra-centrifuged (L-70 Ultracentrifuge Beckman Coulter, Brea, CA, USA) at 20,000 rpm for 10 min at 4 °C. The supernatant was analyzed for carotenoid content, while the pellet was discarded. Carotenoid bioaccessibility values were calculated as the ratio between the carotenoid concentration in the supernatant (in the micellar fraction) of the intestinal fraction and its initial concentration in the emulsion prior to in vitro digestion (Equation (1)).

Bioaccessibility (%) = (Carotenoid content supernatant/Carotenoid content emulsion) × 100 (1)

#### 2.10.2. Carotenoid Extraction from In Vitro Digestion Phases

Carotenoids were extracted from the digesta according to the method described by Lara-Abia et al. [7]. The digesta was extracted with 15 mL of acetone and placed in an ultrasound bath for 15 min at 25 °C. Then, samples were centrifuged at 9000 rpm for 10 min at 4 °C, and the supernatant was recovered. The pellet was re-extracted twice with acetone and one last time with methanol. Afterward, in a separation funnel, 30 mL of petroleum ether/diethyl ether (1:1; *v/v*) was added to the combined supernatants. The organic phase was recovered and dried with anhydrous sodium sulfate (approx. 2.5 g), filtered, and evaporated until dry in a rotavapor at 30 °C. The sample was made up to 2 mL with MeOH:MTBE:H_2_O (45.5:52.5:2, *v/v/v*), filtered through a 0.45 μm filter, and analyzed through HPLC.

Carotenoids were extracted from the supernatants (micellar fraction) of the digestive phases according to the method described by Petry and Mercadante [23], with modifications. Each supernatant obtained after ultra-centrifugation was extracted with 20 mL of diethyl ether by mixing with a vortex and centrifuging at 14,000 rpm for 10 min at 4 °C in an L-70 Ultracentrifuge (Beckman Coulter, Indianapolis, IN, USA). Then, the supernatant (micellar fraction) was placed in a separation funnel, 20 mL of diethyl ether was added, and the mixture was shaken vigorously. The organic phases were recovered, dried with anhydrous sodium sulfate, filtered, and vacuum-concentrated to be analyzed through HPLC.

### 2.11. Statistical Analysis

All experiments were carried out in duplicate, and three determinations of each analysis were performed on each parameter to obtain mean values. Data are expressed as the mean ± standard deviation. Significant differences were calculated through one-way analysis of variance (ANOVA) and a post hoc Tukey’s test (*p* < 0.05). Student´s *t*-test was used to compare means of carotenoid bioaccessibility values and between the parameters (viscosity, particle diameter, and ζ- potential) of the soybean oil and sunflower oil emulsions. Statistical analyses were performed with IBM SPSS Statistics 23.0 (IBM Corp., Armonk, NY, USA).

## 3. Results and Discussion

### 3.1. O/W Papaya Carotenoid Microemulsion Optimization Process

**Figure 1:**
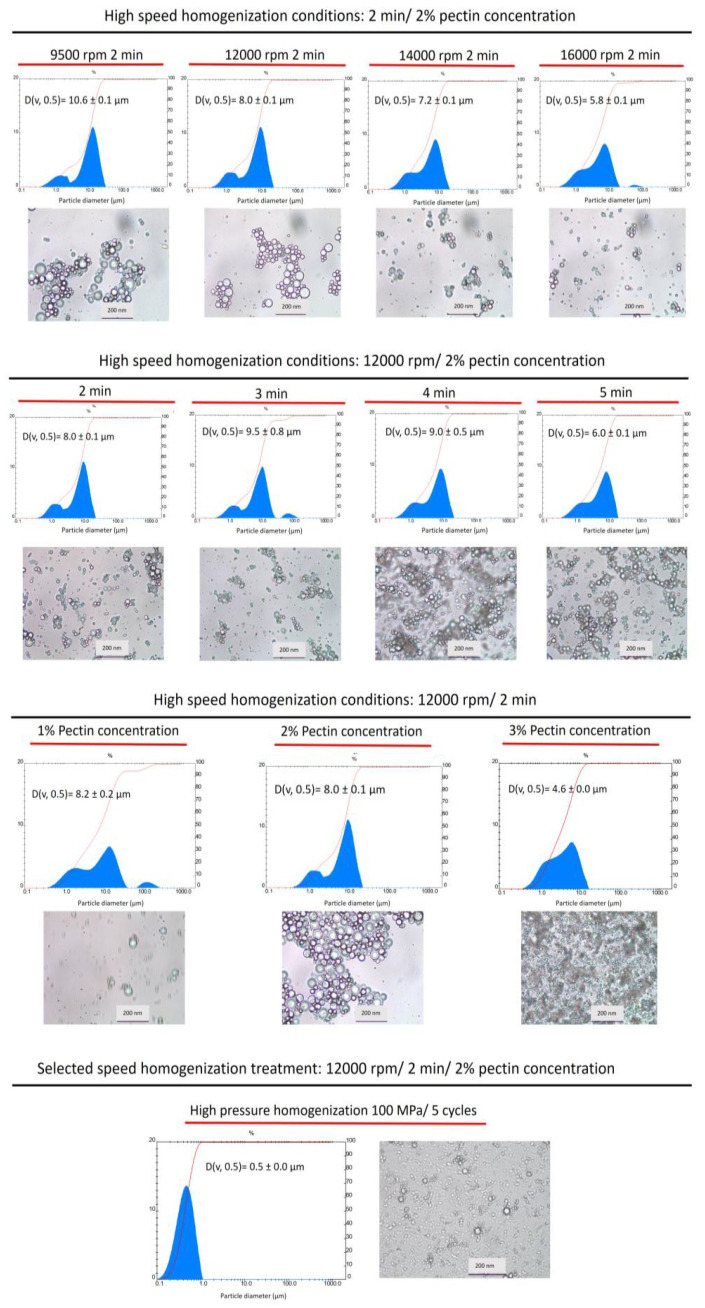
Particle size distribution and optical microscopy images of sunflower oil O/W emulsions obtained using different homogenization speeds (9500, 12,000, 14,000, and 16,000 rpm), time periods (2, 3, 4, and 5 min), and pectin concentrations (1%, 2%, and 3%). Scale bar = 200 µm.

Different ultra-homogenization conditions were evaluated using the Ultraturrax equipment to obtain the optimum coarse emulsion. Vegetable oils (sunflower and soybean oils) enriched with papaya peel direct extract (7 mg carotenoid/g oil, *w/w*) were used to optimize the O/W microemulsions. The carotenoid composition of this papaya peel extract is shown in Table S2 (Supplementary Materials), and the contents of different carotenoids in the papaya peel extract are displayed in Table S3. To achieve better conditions for procuring stable O/W emulsions, a two-step process was assayed, following the procedure of Teixé-Roig et al. [15] with some modifications. In the first stage of process validation, the papaya carotenoid extract dissolved in sunflower oil (7 mg carotenoid/g oil, *w/w*) was used to optimize the procurement of O/W coarse emulsion. The oil-in-water ratio was 1:24 (4% lipid phase and 96% aqueous phases). High-speed homogenization (rpm) was the initial parameter evaluated. No significant differences were found when using speeds of 9500, 12,000, 14,000, and 16,000 rpm in terms of the particle size and viscosity of the obtained O/W emulsions (Table 1). Therefore, 12,000 rpm was the speed selected for this step, since the distribution particle graph and the optical microscopy image showed good uniformity in the generated O/W emulsions (Figure 1). Then, the high-speed homogenization was evaluated in terms of the time taken (2, 3, 4, and 5 min). In this case, since no differences in the physicochemical characteristics (particle diameter, viscosity, ζ-potential, and in particle distribution and microscopy graphs) of the O/W emulsions were found, a time of 2 min was selected to continue with the second step of obtaining stable O/W emulsions. Finally, three different pectin concentrations (1%, 2%, and 3%) were assayed to formulate different O/W coarse emulsions. The smallest particle diameter (2.7 ± 0.1 μm) was obtained using a 2% pectin concentration, in comparison with 1% (8.2 ± 0.2 μm) and 3% (4.6 ± 0.0 μm).

No differences were found among the viscosity values (mPa.s) of the O/W coarse emulsions prepared with different concentrations of pectin, 1% (5.4 ± 0.0), 2% (5.4 ± 0.0), and 3% (5.5 ± 0.0), after the high-speed homogenization of the lipid phase at 12,000 rpm for 2 min (coarse emulsion) when coconut oil was used (Table 2).

After the selection of the optimum conditions (12,000 rpm for 2 min with 2% pectin concentration), Tween 20 was used as a second low-molecular-size emulsifier to formulate the coarse emulsion. Next, the final O/W emulsion was obtained using high-pressure homogenization (HPH) treatment at 100 MPa/5 cycles (see Materials and Methods section). The conditions selected for this last step were established according to the study reported by Teixé-Roig et al. [15] for the encapsulation of standard β-carotene, but in the present work, high-pressure homogenization (HPH) equipment was used instead of a microfluidizer. After HPH treatment, a particle size of 0.5 ± 0.0 μm, a viscosity of 7.3 ± 0.2 mPa.s, and a ζ-potential of −26.8 ± 0.2 were obtained in the corresponding final emulsion (Table 1). The particle distribution graph of this formulation showed great uniformity among the particles, and in the light microscopy image (Figure 1), a reduction in the emulsion size as well as pectin aggregation can be observed, possibly due to temperature increase during the HPH process (2.4 °C/100 bar).

Once the optimization of the O/W emulsion process was established for long-chain fatty acid vegetable oils (soybean and sunflower oils), coconut oil (medium-chain triglyceride) was assayed to formulate similar O/W emulsions (Table 2). The objective was to evaluate the possible differences in particle diameter, viscosity, ζ-potential, particle distribution, and stability of the final coconut oil O/W emulsions, compared to the long-chain triglyceride oil (soybean oil and sunflower oil) O/W emulsions. Table S2 shows the composition of the fatty acids in the assayed vegetable oils [18] to formulate the papaya carotenoid O/W emulsions. During the homogenization process, the high pressure applied pushes the emulsion through the small annular space, where the pressure is transformed into high velocity, generating extreme turbulence and cavitation, reducing the emulsion particle size [24].

In the coconut O/W emulsions, as the pectin concentration increased, the ζ-potential increased as follows: −35.0 ± 2.5 mV (1% pectin), −24.8 ± 0.7 mV (2% pectin), and −15.6 ± 0.2 mV (3% pectin). The opposite effect occurred in terms of the particle diameter of O/W coarse emulsions, with the highest particle diameter (v, 0.5) at a pectin concentration of 1% (11.2 ± 0.2 μm), followed by 6.7 ± 0.0 μm at a 2% pectin concentration and 3.1 ± 0.1 μm (the smallest) at a 3% pectin concentration. These results show that stable and small-particle coarse emulsions were obtained when the concentration of pectin was 3%. However, after the HPH treatment at 100 MPa for five cycles, similar values of viscosity, particle diameter, and ζ-potential were observed in the final emulsions for all pectin concentrations (Table 2). These final O/W coconut oil emulsion data were also not significantly different to those observed for the O/W final emulsions formulated with long-chain triglycerides (soybean and vegetable oils) (Table 1).

**Figure 2:**
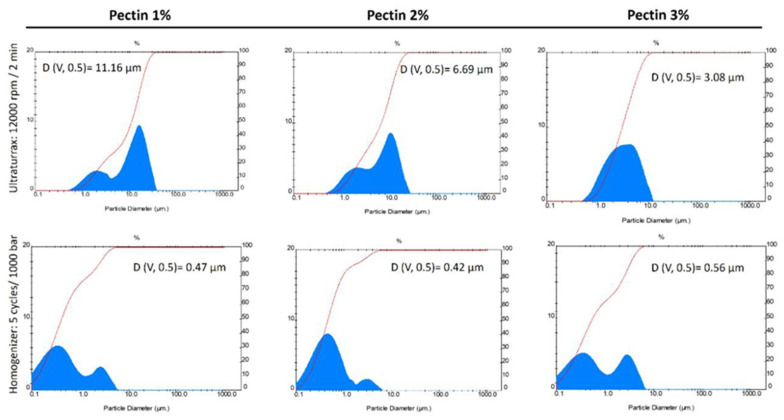
Particle distribution of coconut oil (MCT) emulsions formulated with high-speed homogenization at 12,000 rpm for 2 min (upper row) with 1%, 2%, and 3% pectin; the same emulsions after treatment with high-pressure homogenization (HPH) at 1000 bar (100 MPa) for 5 cycles (lower row).

**Figure 3:**
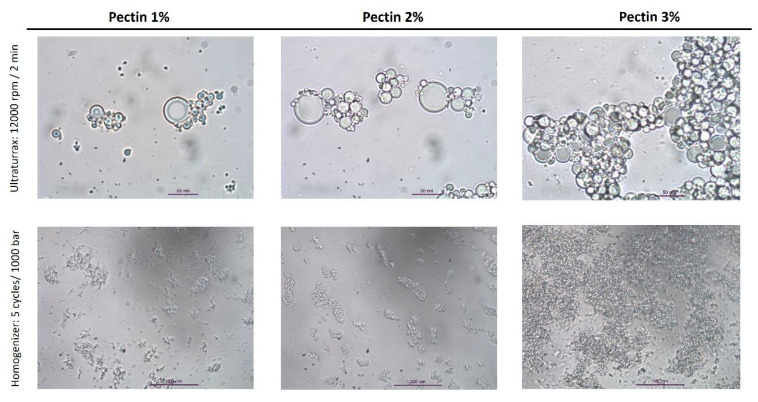
Optical microscopy of MCT (coconut oil) emulsions formulated using high-speed homogenization (Ultraturrax) at 12,000 rpm for 2 min (upper row) with 1%, 2%, and 3% pectin; the same emulsions after treatment with high-pressure homogenization (HPH) at 1000 bar (100 MPa) for 5 cycles (lower row). Scale bars = 50 μm for images in the upper row and 200 μm for images in the lower row.

In contrast, regarding the particle size distribution (Figure 2) and the optical microscopy images (Figure 3) of the coconut (MCT) O/W final emulsions, a notable coalescence effect between particles was observed, and instability became evident in high-pressure homogenization (HPH)-treated emulsions. The coalescence in the O/W emulsions could be due to different factors such as the required solid particles in the dispersed oil phase, the formation of irregular clusters, and the increased aggregation rate [24]. In the case of coconut (MCT) O/W emulsions, a greater electrostatic repulsion instead of a steric repulsion between the oil droplets and pectin molecules might be the factor leading to emulsion stability. Consequently, the particles tend to aggregate and produce the observed coalescence. For this reason, the formulation using coconut oil (MCT) was discarded before continuing the study for improved stability and bioaccessibility of papaya carotenoids using in vitro gastrointestinal assays.

### 3.2. Physicochemical Characterization

#### 3.2.1. Particle Size

**Figure 4:**
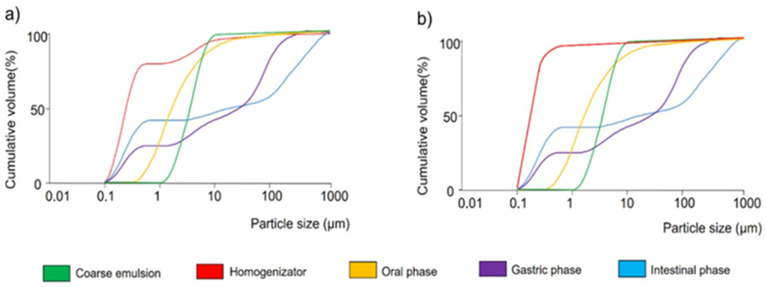
Particle size distribution of (**a**) soybean emulsions (coarse emulsion and HPH-treated emulsion) and (**b**) sunflower emulsions in different phases of the in vitro gastrointestinal digestion.

The coarse soybean oil and sunflower oil emulsions (before treatment with HPH) presented a higher mean particle size (6.6 ± 0.0 μm and 5.1 ± 0.0 μm, respectively) (Figure 1). After the high-pressure homogenization process, the particle sizes reduced to 0.6 ± 0.0 for the soybean oil emulsion and 0.4 ± 0.0 μm for the sunflower oil emulsion (Table 3). These differences observed in the particle sizes showed that in addition to reducing the particle size, the homogenization process created a microemulsion less polydisperse compared to the coarse emulsion (Figure 4). However, only particle sizes corresponding to microemulsions were obtained. Meanwhile, authors such as Teixé-Roig et al. [15] obtained lower emulsion particle sizes (439.3 ± 23.3 nm) using a corn oil O/W, 2% pectin, and a microfluidification process.

During emulsification, only a small proportion of the pectin actually adsorbs on oil droplets to form a protective layer, which decreases the oil/water interfacial tension, reducing the emulsion droplet size, as observed in the present study. In the literature, it is reported that pectin, a plant cell wall polysaccharide, is a natural multifunctional ingredient that imparts textural and rheological properties to a wide range of food systems. The emulsion-stabilizing properties of pectin are controlled by the homogalacturonan (HG) domain and the neutral sugar side chains of the rhamnogalacturonan-I (RGI) structural element. However, the neutral sugar side chains might obstruct the accessibility of pectin hydrophobic species to the oil/water interface, hampering emulsification. In addition, the contribution of HG to emulsion stabilization might depend on the polymer HG:RGI ratio. Hence, the influence of the structural features of pectin on the polymer-emulsifying potential is yet to be fully unraveled, as identified in a published review [25]. The presence of Tween 20 together with pectin in the O/W emulsions may help reduce the particle size, since this surfactant has a lower molecular weight than pectin and could shift faster toward the interface in a competitive manner that may lead to small particle sizes, as reported previously [10]. The viscosity of the sunflower oil emulsion was significantly (*p* < 0.05) higher (6.2 ± 0.1 mPa.s) than that of the soybean oil emulsion (5.3 ± 0.0 mPa.s). This difference could enhance the reduction in the particle size by increasing the disruptive shear stress [26]. However, other authors [13] reported a higher viscosity, of 19.77 ± 0.26 mPa.s, when an O/W emulsion was formulated with corn oil and 2% of pectin to encapsulate standard β-carotene. This difference could be due to the different vegetable oil composition in fatty acids and to the technology employed to obtain the nanoemulsion, because they used microfluidizer equipment and in the present study, a high-pressure homogenizator was employed. These two pieces of equipment have different technical characteristics, and it is the interaction chamber that could influence the reduction in particle sizes in O/W emulsions.

#### 3.2.2. Electrical Charge

**Figure 5:**
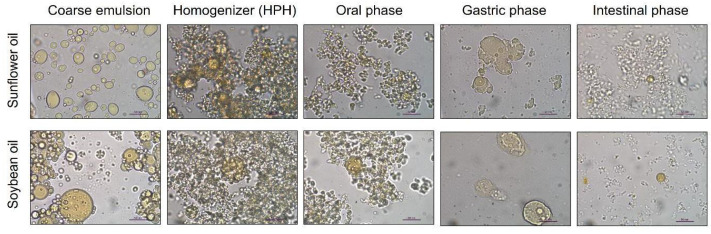
Images of the coarse emulsions and final emulsions in different phases of the in vitro digestion. Scale bar = 50 µm.

The electrical charge of the soybean emulsion was −25.7 ± 1.0 mV and that of the sunflower emulsion was −26.5 ± 1.3 mV, which are less negative values than the values before the homogenization process (−29.1 ± 1.6 mV for coarse soybean emulsion and −35.7 ± 2.9 mV for coarse sunflower emulsion) (Figure 5). These emulsions were formulated with Tween 20, which is a non-ionic surfactant. Therefore, it is expected that there will be no negative charge. Thus, the negative charges may be related to the preferential absorption of OH- species from water to the oil–water interface [26].

#### 3.2.3. Encapsulation Efficiency

Table 3 presents the encapsulation efficiencies obtained in the present study.

These values were calculated using the total carotenoid content analyzed from the O/W microemulsions related to the content of each carotenoid in the papaya peel extract. The encapsulation efficiency was 91–92% for both O/W microemulsions. These data indicate that the formulations were effective in encapsulating the direct extract of papaya peel carotenoids. Relating to the main individual papaya carotenoid encapsulation values, a range between 85 and 97% was observed, with higher values for the hydrocarbon carotenoids, i.e., (*all*-*E*)-β-carotene (95%) and (*all*-*E*)-lycopene (97%), and lower values for the xanthophylls, i.e., (*all*-E)-β-cryptoxanthin (85%) and (*all*-*E*)-β-cryptoxanthin laurate (88%). In the literature, there are no data on the encapsulation efficiency of individual carotenoids from encapsulated direct papaya extracts, but in published studies about the encapsulation of individual carotenoid standards or of those isolated from fruit by-products, the encapsulation efficiencies reported range from 76.5% for β-lycopene extract encapsulated in gelatin and poly(γ-glutamic acid) as carriers [27] to 51.7% for β-carotene encapsulated in lipid microparticles stabilized with hydrolyzed soy protein isolate [28]. In the present work, the O/W microemulsions were efficient in encapsulating the main carotenoids from the papaya by-product (peel).

### 3.3. Behavior of the Papaya Carotenoid O/W Emulsions during In Vitro Gastrointestinal Digestion

#### 3.3.1. Particle Size

**Figure 6:**
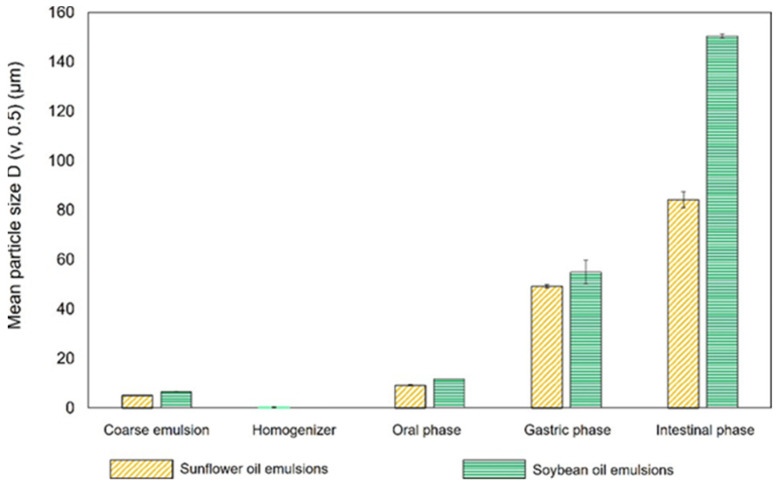
Mean particle size of O/W soybean and sunflower final emulsions (coarse emulsion and HPH-treated emulsion) in different phases of the in vitro gastrointestinal digestion.

Figure 4 and Figure 5 show the mean particle size and particle size distribution, respectively, in the phases of in vitro gastrointestinal digestion. In the oral phase, no significant differences were observed in the mean particle sizes and particle size distribution between the O/W sunflower and soybean emulsions. In the gastric and intestinal phases, the particle size distribution became more polydisperse, indicating that many large particles were formed. This could be due to the use of Tween 20, which has been reported to provide greater stability in in vitro digestion compared to other surfactants, such as proteins or lecithin, which are less effective against flocculation [28]. Optical microscopy images of the in vitro gastrointestinal phases (Figure 5) showed flocculence and coalescence between particles, mainly in the gastric phases of both types of studied O/W soybean and sunflower emulsions, which could be the reason why the mean particle size increased significantly during these phases. Figure S4 (Supplementary Materials) shows the images taken with confocal microscopy of the (a) soybean and (b) sunflower final emulsions after they were processed through high-pressure homogenization (HPH) at 100 MPa for five cycles, where different distributions can be observed. In addition, the pectin present in the aqueous phase could promote this aggregation between molecules, generating gel-like pectin, namely Ca^2+^ cross-linkings with calcium ions present in the gastric fluids, which are clustering oil droplets [13]. In the intestinal phase, these oil droplets are digested by the intestinal enzymes. The noticeable increase in particle size at this stage could be related to the formation of mixed micelles. Figure 4 and Figure 6 show the changes in the mean particle size of the O/W soybean and sunflower final emulsions in different phases of the in vitro gastrointestinal digestion. There could be some undigested oil droplets and some coalescence between molecules, since the interface stabilizers during the intestinal phase may be altered, losing their efficiency [29]. Images taken with a confocal microscope of the (a) soybean and (b) sunflower final emulsions after they were processed through high-pressure homogenization (HPH) at 100 MPa for five cycles are displayed in Figure S4 (Supplementary Materials).

#### 3.3.2. Electrical Charge

**Figure 7:**
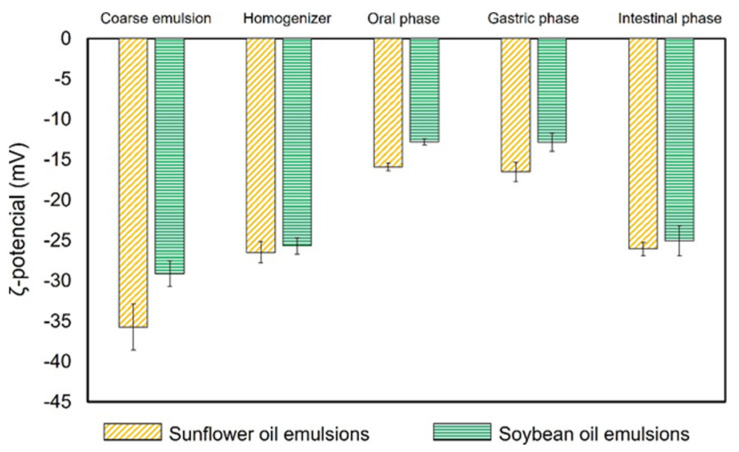
ζ-potential values of O/W carotenoid coarse and final emulsions in different phases of the in vitro gastrointestinal digestion.

During the oral and gastric phases, the ζ-potential remained constant for both the O/W emulsions (sunflower and soybean) but less negative than that for the coarse and the final emulsions before digestion (Figure 7). In the gastric phase, the presence of positive ions (Ca^2+^, K^+^, and H^+^) could be responsible for the significant decrease in the negative electrical charge values. In addition, since most of the pectin carboxyl groups are protonated in the gastric phase, they cannot interact with the surrounding ions. In the intestinal phase, for both the O/W carotenoid soybean and sunflower emulsions, the negativity in the electrical charge increased almost to the same level as that for the coarse and final emulsions before digestion.

As Chang and McClements [30] reported, since the pH is neutral in the intestinal phase, the lipid droplets and the pectin present produced negative charges, creating repulsion between them, hindering the adsorption of the pectin on the interface. However, due to the high presence of negative molecules in this phase (e.g., bile salts), this effect was covered and was difficult to measure.

#### 3.3.3. Carotenoid Stability and Bioaccessibility in O/W Microemulsions

The stability and bioaccessibility of the most abundant carotenoids ((*all*-*E*)-β-cryptoxanthin, (*all*-*E*)-β-cryptoxanthin laurate, (*all*-*E*)-β-carotene, and (*all*-*E*)- lycopene) found in the O/W papaya by-product carotenoid emulsions are displayed in Table 4.

Figure S1 (Supplementary Materials) shows the HPLC chromatograms of the carotenoids in the digesta fractions (carotenoids in each phase of the digestion) of all carotenoids (hydrocarbon carotenoids, xanthophylls, and xanthophyll esters) in the vegetable oil extracts, in the O/W formulated emulsions, and in the carotenoid O/W emulsions before the in vitro gastrointestinal digestion assay and during the in vitro digestion. The content of each main carotenoid in the digesta fraction provides information about the stability of the carotenoids during in vitro gastrointestinal digestion. Figure S2 (Supplementary Materials) shows the carotenoid compounds identified during the in vitro digestion of O/W soybean and sunflower emulsions in the micellar fractions (carotenoids that were in the micellar fraction of each digestion phase). The content of each carotenoid in the micellar fraction of the intestinal phase is the value used for calculating the bioaccessibility of carotenoids (see the Materials and Methods section). Table S2 shows the characterization of the carotenoids identified in the extracts of papaya by-product (peel) as reported by Lara-Abia et al. [7].

Regarding the obtained results, the bioaccessibility of (*all*-*E*)-β-cryptoxanthin in the soybean (15%) and sunflower (15%) O/W emulsions was the same (Table 4). However, significant differences (*p* < 0.05) were observed for its major xanthophyll ester, (*all*-E)-β-cryptoxanthin laurate, showing higher bioaccessibility in the O/W sunflower emulsion (7.2 ± 0.9%) than in the O/W soybean emulsion (3.5 ± 0.1%). With respect to (*all*-E)-β-carotene, higher bioaccessibility was observed in both the O/W soybean (17.9 ± 0.7%) and sunflower (18.4 ± 0.4%) emulsions compared to its bioaccessibility in a previous study of the in vitro digestion of un-encapsulated extracts [7]. (*all*-E)-Lycopene showed the highest bioaccessibility among all the carotenoids of the extracts of papaya encapsulated by O/W emulsions. Bioaccessibility values of 64 ± 4% for the O/W soybean and of 71 ± 4% for the sunflower emulsions were obtained for (*all*-E)-lycopene, possibly because this hydrocarbon carotenoid is highly soluble in the vegetable oils employed to formulate the O/W emulsions. The same reason may be applicable to the high (all-*E*)-β-carotene bioaccessibility, since hydrocarbon carotenoids are more hydrophobic than xanthophylls and xanthophyll esters. Zhao et al. [31] reported lower (all-*E*)-lycopene bioaccessibility (around 25%) in sesame oil and linseed oil nanoemulsions and bioaccessibility of 18% in walnut oil nanoemulsions when lactoferrin was used as an emulsifier, showing a similar trend with their stability. In addition, the results obtained in the present study showed higher bioaccessibility values for carotenoids in the O/W sunflower emulsion than for those in the O/W soybean emulsion (Table 4), possibly because the O/W sunflower emulsion presented smaller particle size (higher surface area), which could improve oil digestibility and the transfer of carotenoids to micelles during the gastrointestinal digestion (intestinal) phase.

The formation of pectin gels during the gastric phase may have a protective effect on carotenoids, enhancing the bioaccessibility of the carotenoids liberated from the O/W emulsions, facilitating their transport toward the intestinal phase. Other factors, such as the degree of oil unsaturation of the fatty components of vegetable oils and the carotenoid nature, affect their micellarization and bioaccessibility [11]. In this sense, monounsaturated fatty acids, such as the oleic acid present in sunflower oil, might be more hydrophobic in comparison to polyunsaturated fatty acids present in soybean oil (mainly composed of linoleic acid and linolenic acid), leading to better solubilization of hydrophobic carotenoids, such as β-cryptoxanthin and β-cryptoxanthin laurate, into the micelles. The stability (bioactive content) of (*all*-E)-β-cryptoxanthin, (*all*-E)-β-cryptoxanthin laurate, (*all*-E)-β-carotene, and (*all*-E)-lycopene during the in vitro gastrointestinal digestion of the soybean and sunflower emulsions is shown in Figure S3 (Supplementary Material), and the carotenoid content is shown in Table 4. A recently published study [3] reported low bioaccessibility of papaya carotenoid from the fruit pulp (edible portion) of the Sweet Mary papaya variety. In this previous study [3], the (*all*-E)-β-cryptoxanthin bioaccessibility value was ≈ 3.4 ± 0.1%, while in the present study, when the papaya extract was encapsulated by O/W emulsions, the bioaccessibility of this carotenoid showed an increase of more than 5 times (15.4%) for this xanthophyll for both formulated O/W emulsions (Table 4). Regarding the bioaccessibility of the most abundant xanthophyll ester of papaya peel extracts, (*all*-E)-β-cryptoxanthin laurate, in the encapsulated extract O/W emulsions, an increase of 3 times was observed with values of 3.5% for the O/W soybean emulsion and 7.2% for the O/W sunflower emulsion. Similar results were observed for the hydrocarbon carotenoids, (*all*-E)-β-carotene and (all-E)-β-lycopene. In general, the encapsulation of papaya by-product extract by O/W emulsions significantly improved the bioaccessibility of the carotenoids, with the greatest bioaccessibility for (*all*-E)-β-lycopene (with bioaccessibility values up to 63.8% in the O/W soybean emulsion and 71.1% in the O/W sunflower emulsion), while the bioaccessibility of this carotenoid in non-encapsulated papaya carotenoid extract was only 0.3%. Regarding (*all*-E)-β-carotene, the obtained bioaccessibility values for the carotenoid papaya extract encapsulated by O/W emulsions showed an improvement of up to 17.3% (soybean oil emulsion) and 17.8% (sunflower oil emulsion), while the bioaccessibility of (*all*-E)-β-carotene from the un-encapsulated papaya extract was only 0.6% [17]. However, the bioaccessibility values for (*all*-E)-β-carotene encapsulated by O/W emulsions in the present study using a 2% pectin concentration were lower than those obtained by Teixé-Roig et al. [14], who reported a bioaccessibility of ≈36% for encapsulated extract. This difference could be due to the use of different vegetable oils to produce the coarse emulsion: they used corn oil instead of the sunflower and soybean oils that were employed here to encapsulate direct carotenoid extract from the papaya by-product. However, in both studies, a significant improvement was observed in the bioaccessibility of (*all*-E)-β-carotene when O/W microemulsions were used.

## 4. Conclusions

The process for optimizing the formulation of O/W emulsions using soybean oil, sunflower oil, and coconut oil enriched with carotenoids from a papaya by-product (peel) was studied using high-speed and high-pressure homogenization processes and pectin. The obtained sunflower O/W microemulsion showed smaller mean particle size, higher negative ζ-potential, and higher viscosity than the soybean O/W microemulsion. The coconut O/W microemulsion was not stable and thus was not used for the digestion assays. Pectin could act as a protective barrier in the formulation of O/W microemulsions, increasing the bioaccessibility of encapsulated carotenoids. A reduction in the size of the particles in the sunflower O/W microemulsion favored the bioaccessibility of the studied carotenoids, with (*all*-E)-β-lycopene having higher bioaccessibility (64% and 71% in the soybean and sunflower O/W emulsions, respectively), followed by (all-E)-β-carotene, which showed similar bioaccessibility in the soybean (18%) and sunflower (18%) O/W emulsions. (*all*-E)-β-cryptoxanthin also had similar bioaccessibility for both the soybean (15%) and sunflower (15%) O/W microemulsions (15%), and (*all*-E)-β-cryptoxanthin laurate was the carotenoid that presented the lowest bioaccessibility values: 4% in the soybean emulsion and 7% in the sunflower emulsion. The bioaccessibility of (*all*-E)-β-carotene and (*all*-E)-β-lycopene also increased up to 17.3% and 63.8% in the soybean O/W microemulsions and 17.8% and 71.1% in the sunflower O/W microemulsions, respectively.

The high degree of unsaturation of sunflower oil and soybean oil fatty acids may promote the formation of small droplets and increase the interface flexibility in the emulsions due to unsaturated linkages. However, it is notable that there is scarce information on the influence of the degree of saturation of the vegetable oil employed to formulate the O/W emulsions on the bioaccessibility of carotenoids during gastrointestinal digestion. For this reason, further in vivo investigations are needed to understand the digestive behavior of O/W emulsions containing carotenoids to assess the real bioavailability of the encapsulated carotenoids from direct extracts.

## Figures and Tables

**Table 1:** Physicochemical characteristics (particle diameter (v, 0.5 μm), viscosity (mPa.s), and ζ-potential (mV)) of O/W sunflower oil emulsions obtained using different high-speed homogenization speeds (9500, 12,000, 14,000, and 16,000 rpm), time periods (2, 3, 4, and 5 min), and pectin concentrations (1%, 2%, and 3%) and of emulsion treated with high-pressure homogenization (HPH) at 100 MPa/5 cycles.

High Speed Homogenization Conditions			Parameter	
		Particle Diameter D (v, 0.5) (μm)	Viscosity (mPa.s)	ζ-Potential (mV)
*Coarse emulsion*	9500 rpm	10.6 ± 0.1 ^b^	21.9 ± 0.2 ^c^	−43.1 ± 1.1 ^c^
2 min/2% pectin concentration	12,000 rpm	8.0 ± 0.1 ^b^	22.0 ± 1.2 ^c^	−41.9 ± 0.6 ^c^
	14,000 rpm	7.2 ± 0.1 ^b^	22.0 ± 1.5 ^c^	−42.0 ± 0.2 ^c^
	16,000 rpm	5.8 ± 0.1 ^b^	21.4 ± 0.9 ^c^	−32.6 ± 1.2 ^b^
	2 min	8.0 ± 0.1 ^b^	14.8 ± 0.3 ^b^	−37.0 ± 0.4 ^b^
12,000 rpm/2% pectin concentration	3 min	9.5 ± 0.8 ^b^	14.9 ± 1.0 ^b^	−35.7 ± 0.2 ^b^
	4 min	9.0 ± 0.5 ^b^	14.4 ± 0.2 ^b^	−33.1 ± 1.8 ^b^
	5 min	6.0 ± 0.1 ^b^	13.2 ± 0.5 ^b^	−31.8 ± 3.1 ^b^
	1% pectin concentration	8.2 ± 0.2 ^b^	5.7 ± 0.3 ^a^	−40.4 ± 1.6 ^c^
12,000 rpm/2 min	2% pectin concentration	8.0 ± 0.1 ^b^	20.9 ± 1.3 ^c^	−36.5 ± 1.4 ^b^
*Final Emulsion*	3% pectin concentration	4.6 ± 0.0 ^b^	48.8 ± 1.9 ^d^	−27.8 ± 0.5 ^a^
HPH treatment at 100 MPa/5 cycles ^1^		0.5 ± 0.0 ^a^	7.3 ± 0.2 ^a^	−26.8 ± 0.2 ^a^

^1^ Final emulsion obtained using high-speed homogenization at 12,000 rpm for 2 min with 2% pectin concentration aqueous phase. The results are expressed as the mean ± standard deviation. Lowercase superscript letters indicate statistically significant differences (*p* < 0.05) between treatments in the same column.

**Table 2:** Physicochemical characteristics (particle diameter (v, 0.5 μm), viscosity (mPa.s), and ζ-potential (mV)) of MCT (coconut oil) emulsions obtained using different pectin concentrations (1%, 2%, and 3%).

	High-Speed Homogenization (12,000 rpm/2 min)			HPH (100 MPa/5 Cycles)		
Parameter	Pectin Concentration			Pectin Concentration		
	1%	2%	3%	1%	2%	3%
Viscosity (mPa.s)	5.4 ± 0.0 ^b^	5.4 ± 0.0 ^b^	5.5 ± 0.0 ^b^	4.8 ± 0.0 ^a^	5.7 ± 0.4 ^b^	5.5 ± 0.0 ^b^
ζ-potential (mV)	−35.0 ± 2.5 ^d^	−24.8 ± 0.7 ^c^	−15.6 ± 0.2 ^a^	−19.8 ± 0.9 ^b^	−18.1 ± 1.2 ^b^	−19.9 ± 1.9 ^b^
Particle diameter D (v, 0.5) (µm)	11.2 ± 0.2 ^d^	8.0 ± 0.1 ^c^	3.1 ± 0.1 ^b^	0.5 ± 0.0 ^a^	0.4 ± 0.0 ^a^	0.6 ± 0.0 ^a^

The results are expressed as the mean ± standard deviation. Lowercase superscript letters indicate statistically significant differences (*p* < 0.05) between 1%, 2%, and 3% pectin in the same row.

**Table 3:** Particle, size, viscosity, ζ-potential, and bioaccessibility of soybean oil and sunflower oil emulsions with a selected pectin concentration of 2%.

Parameter	O/W Soybean Emulsion	O/W Sunflower Emulsion
Particle diameter D (v, 0.5)	0.6 ± 0.0 ^a^	0.4 ± 0.0 ^b^
Viscosity (mPa.s)	5.3 ± 0.0 ^a^	6.2 ± 0.1 ^b^
ζ-potential (mV)	−25.7 ± 1.0 ^a^	−26.5 ± 1.3 ^a^
Encapsulation efficiency (%)	91.0 ± 0.01 ^a^	92.2 ± 0.0 ^a^

The results are expressed as the mean ± standard deviation. Lowercase superscript letters indicate statistically significant differences (*p* < 0.05) between emulsions.

**Table 4:** Major carotenoid stability (μg/g emulsion) and bioaccessibility (%) during oral phase (O), gastric phase (G), and intestinal phase (I) of in vitro gastrointestinal digestion of soybean and sunflower emulsions.

	O/W Soybean Emulsion					O/W Sunflower Emulsion				
Carotenoid		Stability (µg/g Emulsion) during In Vitro Digestion					Stability (µg/g Emulsion) during In Vitro Digestion			
	Oil Extract	Oral	Gastric	Intestinal	Bioaccessibility (%)	Oil Extract	Oral	Gastric	Intestinal	Bioaccessibility (%)
(*all*-*E*)-β- Cryptoxanthin	0.34 ± 0.0 ^b^	0.47 ± 0.0 ^c^	0.26 ± 0.0 ^b^	0.12 ± 0.0 ^a^	15.4 ± 0.4 ^A^	0.23 ± 0.0 ^a^	0.76 ± 0.0 ^b^	0.56 ± 0.0 ^c^	0.33 ± 0.0 ^b^	15.0 ± 0.2 ^A^
(*all*-*E*)-β- Cryptoxanthin laurate	2.60 ± 0.1 ^b^	2.79 ± 0.1 ^b^	2.28 ± 0.0 ^b^	0.82 ± 0.0 ^a^	3.5 ± 0.1 ^A^	1.92 ± 0.0 ^a^	2.36 ± 0.1 ^b^	2.99 ± 0.3 ^b^	0.98 ± 0.0 ^a^	7.2 ± 0.9 ^B^
(*all*-*E*)-β- Carotene	0.82 ± 0.0 ^a^	1.19 ± 0.0 ^b^	1.56 ± 0.0 ^b^	1.57 ± 0.0 ^b^	17.9 ± 0.7 ^A^	0.77 ± 0.0 ^a^	1.37 ± 0.0 ^b^	0.77 ± 0.0 ^a^	0.19 ± 0.1 ^a^	18.4 ± 0.4 ^A^
(*all*-*E*)-β- Lycopene	3.82 ± 0.3 ^b^	7.14 ± 0.1 ^c^	1.58 ± 0.0 ^b^	1.58 ± 0.0 ^b^	64.1 ± 3.7 ^A^	3.80 ± 0.2 ^b^	8.05 ± 0.1 ^c^	3.75 ± 0.0 ^b^	0.94 ± 0.0 ^a^	71.4 ± 3.9 ^B^

The results are expressed as the mean ± standard deviation. Lowercase superscript letters indicate statistically significant differences (*p* < 0.05) in the carotenoid content between emulsions, and uppercase superscript letters indicate statistically significant differences (*p* < 0.05) between the emulsion bioaccessibility values of the carotenoids.

## Data Availability

Data is contained within the article or Supplementary Materials.

## Supplementary Materials

The following supporting information can be downloaded at https://www.mdpi.com/article/10.3390/foods12142654/s1: Figure S1: C30 reversed-phase chromatograms of carotenoids at 450 nm obtained from Sweet Mary papaya (*Carica papaya* L.) peel encapsulated by O/W emulsions in (a) carotenoid-enriched soybean oil (vegetable oil + papaya carotenoid extract (carotenoid μg/g vegetable oil), (b) carotenoid-enriched sunflower oil (vegetable oil + papaya carotenoid extract (μg carotenoid μg/g vegetable oil), (c) O/W soybean emulsion with encapsulated carotenoid extract, (d) O/W sunflower emulsion with encapsulated carotenoid extract, and in the digesta fractions during the in vitro digestion of (e) O/W soybean emulsion and (f) O/W sunflower oil emulsion in the oral phase, (g) O/W soybean emulsion and (h) O/W sunflower oil emulsion in the gastric phase, and (i) O/W soybean emulsion and (j) O/W sunflower oil emulsion in the intestinal phase. Peak identities in Table S1; Figure S2: C30 reversed-phase chromatograms of carotenoids at 450 nm obtained from Sweet Mary papaya (*Carica papaya* L.) peel encapsulated by O/W emulsions in the micellar fractions during the in vitro digestion of (a) soybean microemulsion and (b) sunflower oil microemulsion in the oral phase, (c) soybean microemulsion and (d) sunflower oil microemulsion in the gastric phase, and (e) soybean microemulsion and (f) sunflower oil microemulsion in the intestinal phase. Peak identities in Table S1; Figure S3: Stability of main papaya carotenoids (μg carotenoids/g emulsion) ((all-E)-β-cryptoxanthin, (all-E)-β-carotene, (all-E)-β-cryptoxanthin laurate, and (all-E)-lycopene) in (a) O/W soybean oil and (b) O/W sunflower oil emulsions before and after in vitro gastrointestinal digestion. Non-digested values refer to the content of each carotenoid in the carotenoid-enriched oil extract before encapsulation by O/W emulsions; Figure S4: Images taken with confocal microscope of (a) soybean and (b) sunflower final emulsions after being processed through high-pressure homogenization (HPH) at 100 MPa for 5 cycles. Scale bars are 5 μm long. Table S1: Percentage (%) fatty acid composition of edible vegetable oils (Dubois et al., 2007) [18]; Table S2: Chromatographic identification ^a^ of carotenoids and carotenoid esters from Sweet Mary papaya (*Carica papaya* L.) peel to formulate soybean oil and sunflower oil carotenoid-enriched emulsions. Table S3: Content of individual carotenoids and carotenoid esters in Sweet Mary papaya (*Carica papaya* L.) peel extracts.
