# Supplementary material for: Improvement in the Stability and Bioaccessibility of Carotenoid and Carotenoid Esters from a Papaya By-Product Using O/W Emulsions
Source: Foods. 2023 Jul 10;12(14):2654. doi: 10.3390/foods12142654 (PMC10379124; doi:10.3390/foods12142654)
Supplement: Supplementary file 1 [file foods-12-02654-s001.zip › foods-2437504-supplementary.pdf]

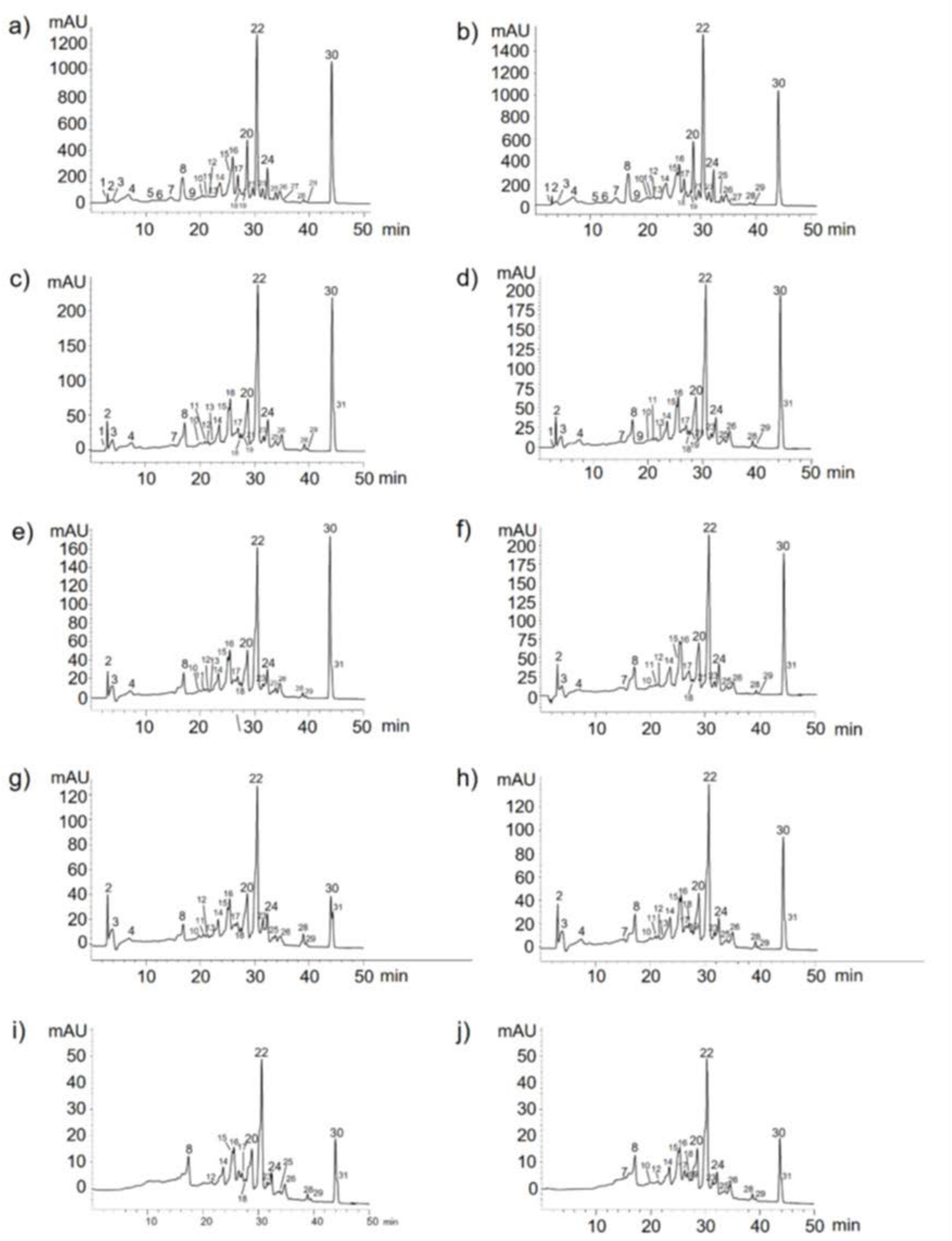

Figure S1. C30 reversed-phase chromatograms of carotenoids at 450 nm obtained from Sweet Mary papaya (Carica papaya L.) peel encapsulated by O/W emulsions in **(a)** carotenoid enriched soybean oil (vegetable oil + papaya carotenoid extract (carotenoid μg/g vegetable oil), **(b)** carotenoid enriched sunflower oil (vegetable oil + papaya carotenoid extract (μg carotenoid μg/g vegetable oil), **(c)** O/W soybean emulsion with encapsulated carotenoid extract, **(d)** O/W sunflower emulsion with encapsulated carotenoid extract, and in the **digesta fractions** during the in vitro digestion of **(e)** O/W soybean emulsion and **(f)** O/W sunflower oil emulsion in the oral phase, **(g)** O/W soybean emulsion and **(h)** O/W sunflower oil emulsion in the gastric phase and in **(i)** O/W soybean emulsion and **(j)** O/W sunflower oil emulsion in the intestinal phase. Peak identities in Table S1.

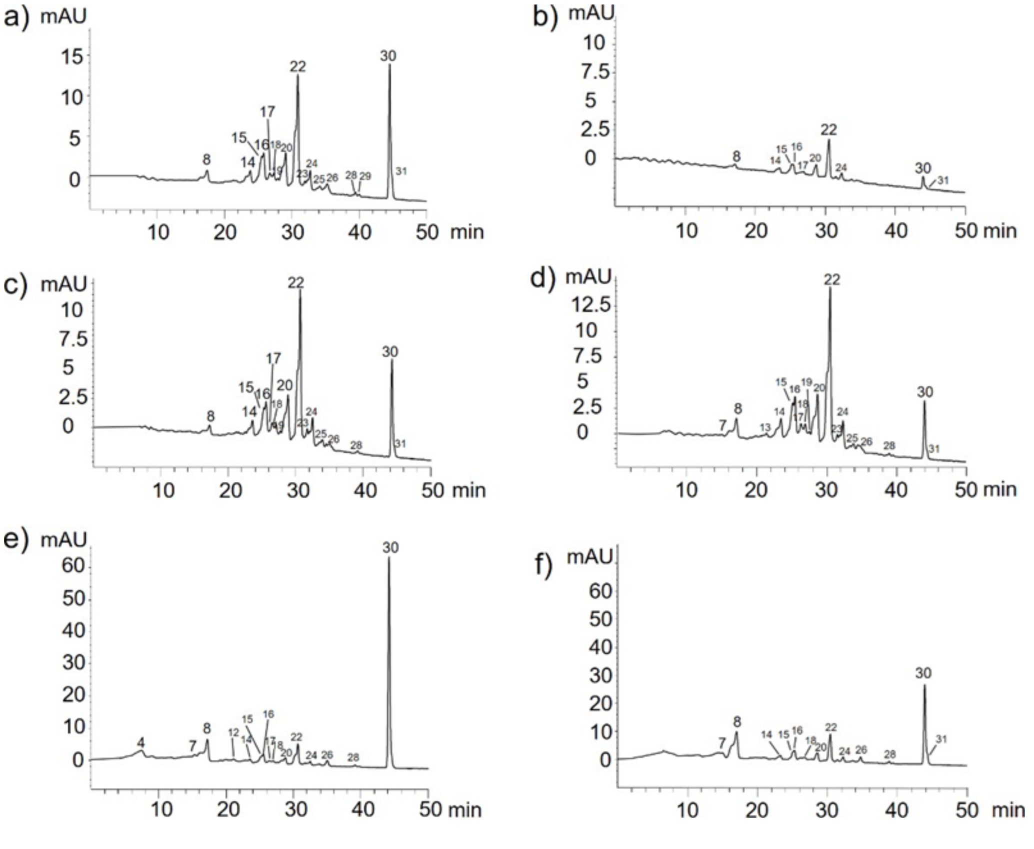

Figure S2. C30 reversed-phase chromatograms of carotenoids at 450 nm obtained from Sweet Mary papaya (Carica papaya L.) peel encapsulated by O/W emulsions in the **micellar fractions** during the in vitro digestion of **(a)** soybean microemulsion and **(b)** sunflower oil microemulsion in the **oral phase**, **(c)** soybean microemulsion and **(d)** sunflower oil microemulsion in the **gastric phase** and in **(e)** soybean microemulsion and **(f)** sunflower oil microemulsion in the **intestinal phase**. Peak identities in Table S1.

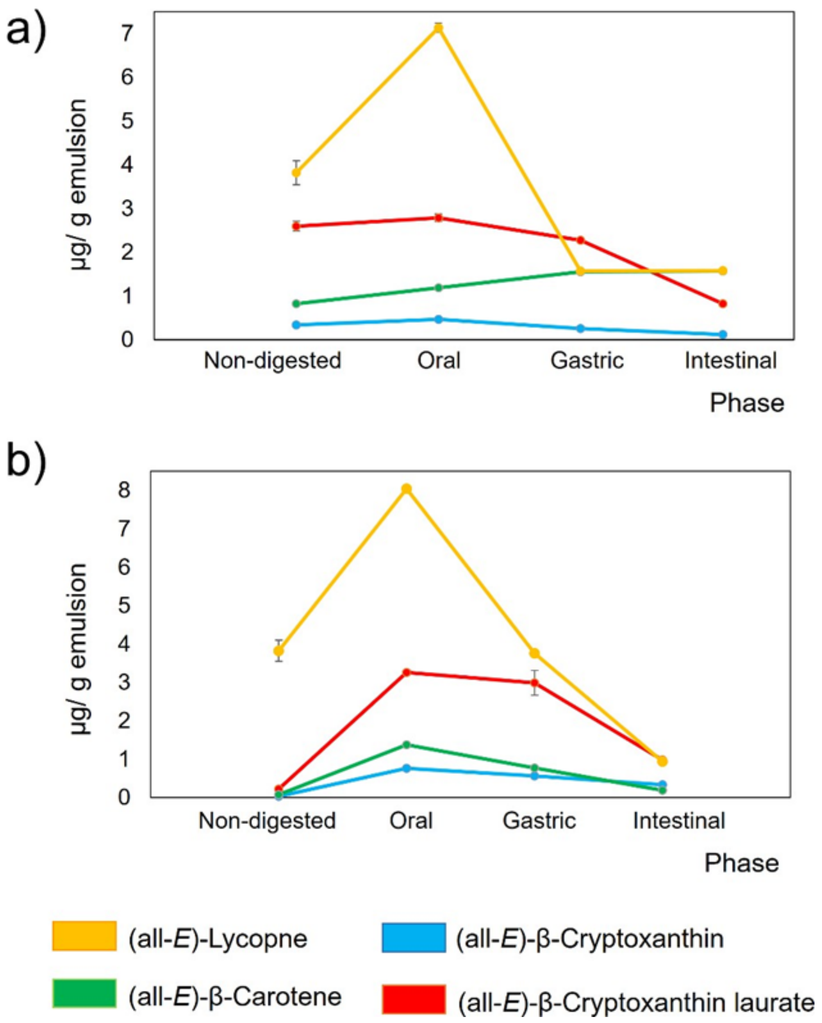

Figure S3. Stability of main papaya carotenoids (µg carotenoids/ g emulsion) ((all-*E*)-β-cryptoxanthin, (all-*E*)-β-carotene, (all-*E*)-β-cryptoxanthin laurate and (all-*E*)-lycopene) in **(a)** O/W soybean oil and **(b)** O/W sunflower oil emulsions before and after in vitro gastrointestinal digestion. Non-digested values are referred to the content of each carotenoid in the carotenoid-enriched oil extract before encapsulation by O/W emulsions.

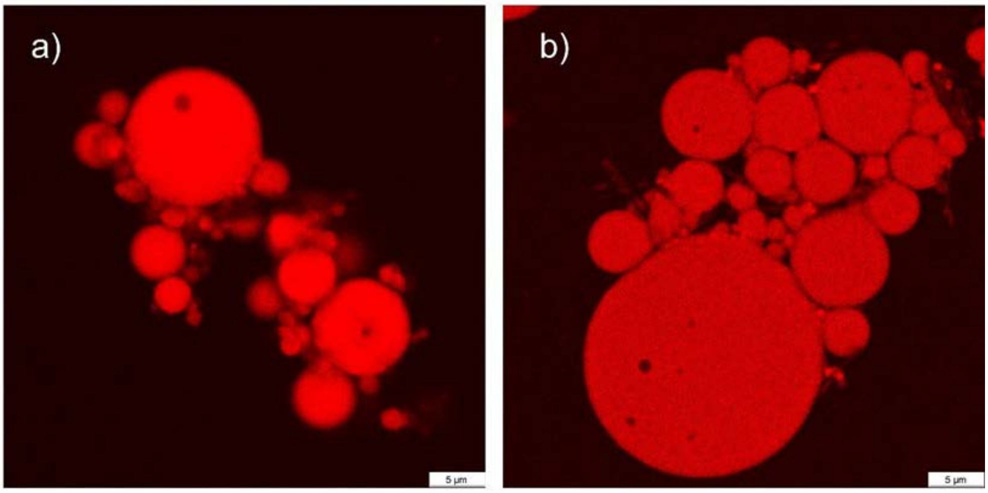

Figure 4S. Images taken with confocal microscope of (a) soybean and (b) sunflower final emulsions after being processed by high pressure homogenization (HPH) at 100 MPa for 5 cycles. Scale bars were 5 µm long.

Table S1. Percentage (%) of fatty acid composition of edible vegetable oils (Dubois et al., 2007) [18].

**Fatty acids**	**Vegetable oils**		
	**Sunflower oil**	**Soybean oil**	**Coconut oil**
Caprylic acid (8:0)	-	-	7.6
Capric acid (10:0)	-	-	6.5
Lauric acid (12:0)	0.5	-	48.2
Myristic acid (14:0)	0.1	0.1	18.5
Palmitic acid (16:0)	6.4	10.8	8.7
Stearic acid (18:0)	4.5	3.9	2.7
Arachidic acid (20:0)	0.3	0.3	0.1
Behenic acid (22:0)	0.8	0.2	-
Lignoceric acid (24:0)	0.2	0.3	-
Palmitoleic acid (16:1 n-7)	0.1	0.2	-
Oleic acid (18:1 n-9)	22.1	23.9	6.0
Eicosenoic acid (20:1 n-9)	0.2	0.1	0.1
Erucic acid (22:1 n-9)	0.1	-	-
Linoleic acid (18:2 n-6)	65.6	52.1	1.8
α-Linolenic acid (18:3 n-3)	0.5	7.8	0.1

From

Dubois, V., Breton, S., Linder, M., Fanni, J. and Parmentier, M. (2007), Fatty acid profiles of 80 vegetable oils with regard to their nutritional potential. Eur. J. Lipid Sci. Technol., 109: 710-732. https://doi.org/10.1002/ejlt.200700040

Table S2. Chromatographic identification[a] of carotenoids and carotenoid esters from Sweet Mary papaya (Carica papaya L.) peel to formulate soybean oil and sunflower oil carotenoid enriched emulsions.

Peak	Rt (min)	Compound identity	HPLC-DAD UV/Vis Absorption Maxima (nm)	%III/II	%Ab/AII	$[M+H]^+$ *m/z*	HPLC/APCI$^+$ MS Fragmentation Pattern (*m/z*)
1	3.4	(13Z)-violaxanthin	326, 416, 440, 470	78	0	601	583 [M + H – 18]$^+$, 565 [M + H – 36]$^+$, 509 [M + H – 92]$^+$, 491 [M + H – 92 – 18]$^+$
2	3.8	(all-*E*)-violaxanthin	414, 438, 468	98	0	601	583 [M + H – 18]$^+$, 565 [M + H – 36]$^+$, 521 [M + H – 80]$^+$
3	7.6	(all-*E*)-neoxanthin	416, 437, 469	99	0	601	583 [M + H – 18]$^+$, 565 [M + H – 36]$^+$, 547 [M + H – 54]$^+$, 521 [M + H – 80]$^+$
4	7.9	(all-*E*)-lutein	(420), 444, 472	62	0	569	551 [M + H – 18]$^+$, 533 [M + H – 36]$^+$
5	12.3	(9Z)-violaxanthin	327, 414, 436, 468	89	n.c.	601	583 [M + H – 18]$^+$, 565 [M + H – 36]$^+$
6	13.7	β-cryptoxanthin-5,6-epoxide	(420), 445, 471	52	n.c.	569	551 [M + H – 18]$^+$, 459 [M + H – 18 – 92]$^+$, 221
7	14.4	(9Z)-α-cryptoxanthin	412, 437, 466	0	0	n.d.	n.d.
8	18.1	(all-*E*)-β-cryptoxanthin	(426), 450, 476	18	n.c.	553	535 [M + H – H2O]$^+$, 461 [M + H - 92]$^+$
9	19.3	α-carotene-5,6-epoxide	(418), 441, 469	10	0	553	535 [M + H – 18]$^+$, 495, 205
10	21.2	(13Z)-β-carotene	337, (414), 436, 464	14	0	537	457 [M + H – 80]$^+$, 445 [M + H – 92]$^+$, 400 [M + H – 137]$^+$, 269 [M + H – 268]$^+$, 177 [M+ H – 360]$^+$, 137 [M + H – 400]$^+$
11	21.8	(all-*E*)-violaxanthin laurate	417, 441, 469	n.c.	0	783	765 [M + H – 18]$^+$, 747 [M + H – 18 – 18]$^+$, 691 [M + H – 92]$^+$, 673 [M + H – 92 – 18]$^+$,583 [M + H – 12:0]$^+$, 565 [M + H – 12:0 – 18]$^+$, 547 [M + H – 12:0 – 18 – 18]$^+$
12	22.1	β-cryptoxanthin-5,8-epoxide	412, 438, 464	50	0	569	551 [M + H – 18]$^+$, 459 [M + H – 18 – 92]$^+$,221
13	22.5	(all-*E*)-ζ-carotene	378, 400, 423	108	0	541	472 [M + H – 69]$^+$, 404 [M + H – 137]$^+$,364,337
14	24.0	(all-*E*)-α-carotene	(420), 445, 470	66	0	537	457 [M + H – 80]$^+$, 413 [M + H – 124]$^+$, 177 [M + H – 360]$^+$, 137 [M + H – 400]$^+$, 123 [M+ H – 414]$^+$
15	25.9	(all-*E*)-lutein-3-O-myristate	401, 426, 472	0	0	n.d.	533 [M + H – 228 – 18]$^+$, 495 [M + H – 228 – 56]$^+$, 459 [M + H – 228 – 92]$^+$, 429, 441
16	26.2	(all-*E*)-β-carotene	(428), 450, 476	16	0	537	457 [M + H – 80]$^+$, 445 [M + H – 92]$^+$, 400 [M + H – 137]$^+$, 269 [M + H – 268]$^+$, 177 [M+ H – 360]$^+$, 137 [M + H – 400]$^+$
17	27.4	(all-*E*)-antheraxanthin myristate palmitate	421, 443, 467	31	0	1033	1015 [M + H – 18]$^+$, 941 [M + H – 92]$^+$, 805 [M + H – 14:0]$^+$, 787 [M + H – 14:0 – 18]$^+$, 771 [M + H – 16:0]$^+$,759 [M + H – 16:0 – 18]$^+$, 549 [M + H – 14:0 – 16:0]$^+$, 531 [M +H– 14:0 – 16:0 – 18:0]$^+$
18	27.9	(all-*E*)-violaxanthin palmitate	416, 441, 469	n.c.	n.c.	839	821 [M + H – 18]$^+$, 803 [M + H – 18 – 18]$^+$, 747 [M + H – 16:0]$^+$, 729 [M + H – 92 – 18]$^+$, 583 [M + H – 256]$^+$, 565 [M + H – 18 – 16:0]$^+$, 547 [M + H – 16:0 – 18 – 18]$^+$
19	29.0	(9Z)-neoxanthin dibutyrate	327, 412, 436, 464	80	16	741	723 [M + H – 18]$^+$, 653 [M + H – 4:0]$^+$, 649 [M + H – 92]$^+$, 635 [M + H – 4:0 – 18]$^+$, 631 [M + H – 92 – 18]$^+$, 565 [M + H – 4:0 – 4:0]$^+$, 547 [M + H – 4:0 – 4:0 – 18]$^+$
20	29.2	(all-*E*)-β-cryptoxanthin caprate	428, 450, 476	n.c.	n.c.	707	615 [M + H – 27]$^+$, 535 [M + H – 100]$^+$, 443 [M + H – 11]$^+$, 442 [M + H – 16]$^+$

21	29.5	(all-*E*)-lutein dimyristate	422, 446, 474	38	0	n.d.	761 M + H − 14:0]$^+$, 669 [M + H − 92]$^+$, 553 [M + H − 14:14:0]$^+$
22	30.0	(all-*E*)-β-cryptoxanthin laurate	421, 451, 478	25	0	735	643 [M + H − 92]$^+$, 535 [M + H − 12:0]$^+$, 479 [M + H − 56 − 12:0]$^+$, 443 [M + H − 92 −12:0]$^+$
23	30.4	(all-*E*)-antheraxanthin-3- O-palmitate	422, 444, 472	n.c.	n.c.	823	805 [M + H − 18]$^+$, 787 [M + H − 18 − 18]$^+$, 731 [M + H − 92]$^+$, 567 [M + H − 16:0]$^+$, 549 [M + H − 16:0 − 18]$^+$, 531 [M + H − 16:0 − 18 − 18]$^+$
24	31.7	(all-*E*)-antheraxanthin laurate myristate	418, 442, 470	33	0	977	959 [M + H − 18]$^+$, 777 [M + H − 12:0]$^+$, 749 [M + H − 14:0]$^+$, 759 [M + H − 12:0 − 18]$^+$, 731 [M + H − 14:0 − 18]$^+$, 549 [M + H − 12:0 − 14:0]$^+$, 531 [M + H − 12:0 − 14:0 −18]$^+$
25	32.1	(all-*E*)-β-cryptoxanthin myristate	424, 448, 476	9	0	763	671 [M + H − 92]$^+$, 535 [M + H − 14:0]$^+$, 443 [M + H − 14:0 − 92]$^+$
26	34.0	(13Z)-lycopene isomer 2	442, 465, 493	0	0	537	481 [M + H − 42]$^+$, 467 [M + H − 35]$^+$, 455 [M + H − 100]$^+$, 427 [M + H − 61]$^+$, 413 [M +H − 88]$^+$, 399 [M + H − 24]$^+$, 387 [M + H − 42]$^+$
27	34.5	(all-*E*)-β-cryptoxanthin palmitate	433, 460, 487	0	0	791	699 [M + H − 39]$^+$, 535 [M + H − 100]$^+$, 443 [M + H − 4]$^+$, 413 [M + H − 46]$^+$
28	38.4	(9Z)-lycopene isomer 4	440, 465, 496	0	0	537	481 [M + H − 11]$^+$, 467 [M + H − 32]$^+$, 455 [M + H − 79]$^+$, 427 [M + H − 48]$^+$, 413 [M +H − 30]$^+$, 399 [M + H − 42]$^+$, 387 [M + H − 38]$^+$
29	38.9	(9′Z)-lycopene isomer 5	413, 439, 465, 496	0	0	537	481 [M + H − 49]$^+$, 467 [M + H − 20]$^+$, 455 [M + H − 100]$^+$, 427 [M + H − 24]$^+$, 413 [M +H − 60]$^+$, 399 [M + H − 21]$^+$, 387 [M + H − 19]$^+$
30	43.1	(all-*E*)-lycopene	418, 443, 471, 502	6	0	537	457 [M + H − 80]$^+$, 413 [M + H − 124]$^+$, 177 [M + H − 360]$^+$, 137 [M + H − 400]$^+$, 121 [M+ H − 416]$^+$
31	43.4	(Z)-lycopene isomer 6	443, 471, 502	0	0	537	481 [M + H − 17]$^+$, 467 [M + H − 32]$^+$, 455 [M + H − 100]$^+$, 427 [M + H − 23]$^+$, 413 [M +H − 61]$^+$, 399 [M + H − 40]$^+$, 387 [M + H − 17]$^+$

R.t.: retention time.

n.c.: %III/II was not calculated because of the poor definition of the UV/Vis spectrum or because it was not detected. n.d.: [M + H]+ or MS/MS fragments were not detected.

The complete characterization of carotenoids and carotenoid esters of papaya cv. Sweet Mary pulp and peel may be found in previous studies (Lara-Abia et al., 2021a).

Table S3. Content in individual carotenoids an carotenoid esters in Sweet Mary papaya (Carica papaya L.) peel extracts

No.[2]	Carotenoid compound	cv. Sweet Mary	
		Direct extract (C)	Saponified extract (SAP)
1	(13Z)-violaxanthin	1.2±0.1[a]	112.7±9.4[c]
2	(all-*E*)-violaxanthin	3.4±0.1[a]	115.3±7.8[b]
3	not identified 1	tr.	tr.
4	(9Z)-neoxanthin	n.d	176.8±2.0[a]
5	(all-*E*)-neoxanthin	n.d	37.5±2.9[a]
6	(all-*E*)-lutein	n.d	72.8±0.9[a]
7	(all-*E*)-zeaxanthin	n.d	61.4±1.1[a]
8	lutein-5,6-epoxide	n.d	47.8±1.3[a]
9	(all-*E*)-antheraxanthin	14.5±0.8[a]	44.4±3.6[b]
10	(9Z)-violaxanthin	6.0±0.2[a]	29.0±1.4[b]
11	(all-*E*)-β-apo-caroten-8′ al (IS[3])	147.0±0.6[a]	133.4±0.5[b]
12	5, 6-epoxy-β-cryptoxanthin	n.d	46.8±0.5[a]
13	(9Z)-α-cryptoxanthin	n.d	10.8±0.4[a]
14	not identified 2	tr.	tr.
15	(all-*E*)-α-cryptoxanthin	2.5±.0.1[a]	16.9±0.8[b]
16	(all-*E*)-β-cryptoxanthin	43.1±1.1[a]	418.9±1.5[b]
17	5,6-epoxy-α-carotene	2.6±0.3[a]	n.d
18	(all-*E*)-luteoxanthin	n.d[a]	n.d
19	(13Z)-α-carotene	14.0±0.1[a]	12.8±0.0[b]
20	(13Z)-β-carotene	3.9±0.1[a]	3.1±0.1[b]
21	(all-*E*)-violaxanthin laurate	11.3±1.2[a]	n.d
22	5,8-epoxy-β-cryptoxanthin	7.7±0.7[a]	n.d
23	(all-*E*)-ζ-carotene	4.2±0.3[a]	1.6±0.5[b]
24	5,8′-epoxy-β-cryptoxanthin	28.6±0.3[a]	13.4±0.2[b]
25	(all-*E*)-α-carotene	75.0±0.0[a]	62.1±0.1[b]
26	(9Z)-α-carotene	3.2±0.2[a]	2.4±0.1[b]
27	(9Z)-violaxanthin laurate	50.9±2.5[a]	n.d
28	(all-*E*)-lutein-3-O-myristate	168.7±0.1[a]	n.d

29	(all-*E*)-β-carotene	164.5±6.6[a]	159.2±0.7[b]
30	(9Z)-β-carotene	5.9±0.1[a]	5.1±0.1[b]
31	(all-*E*)-violaxanthin dimyristate	36.0±2.6[a]	n.d
32	(all-*E*)-antheraxanthin myristate palmitate	43.2±1.6[a]	n.d
33	(all-*E*)-violaxanthin palmitate	6.8±0.7[a]	n.d
34	(9Z)-neoxanthin dibutyrate	7.5±0.3[a]	n.d
35	(all-*E*)-β-cryptoxanthin caprate	81.8±1.5[a]	n.d
36	(9Z)-violaxanthin myristate palmitate	n.d	n.d
37	(all-*E*)-lutein dimyristate	60.3±4.0[a]	n.d
38	(all-*E*)-β-cryptoxanthin laurate	174.9±10.4[a]	n.d
39	(all-*E*)-antheraxanthin-3-O palmitate	n.d	n.d
40	(all-*E*)-antheraxanthin laurate myristate	21.9±2.2[a]	8.2±0.6[b]
41	(all-*E*)-β-cryptoxanthin myristate	18.6±1.6[a]	4.6±0.3[b]
42	(Z)-lycopene isomer 1	13.8±0.6[a]	12.0±0.5[b]
43	(all-*E*)-β-cryptoxanthin palmitate	13.9±0.4[a]	5.5±0.0[b]
44	(13Z)-lycopene isomer 2	112.6±0.1[a]	98.3±2.7[b]
45	(13′Z)-lycopene isomer 3	22.4±0.2[a]	16.3±0.3[b]
46	(9Z)-lycopene isomer 4	26.3±0.5[a]	22.1±0.7[b]
47	(9′Z)-lycopene isomer 5	12.3±0.3[a]	9.7±0.7[b]
48	(all-*E*)-lycopene	378.2±5.4[a]	342.9±4.3[b]
49	(Z)-lycopene isomer 6	22.6±0.6[b]	20.4±0.6[b]
	Total free xanthophylls	109.0.±4.0[a]	1204.5±33.7[b]
	Total xanthophyll esters	695.8±29.5[a]	18.2±1.0[b]
	Total hydrocarbon carotenoids	859.0±15.4[a]	767.9±11.3[b]
	Total carotenoids	1663.7±48.9[a]	1990.7±46.1[b]

tr. traces; n.d. not detected (detection limit 0.08 μg/g).

Results are expressed as the mean±standard deviation of duplicate analysis (n = 2) of samples from freeze-dried papaya pulp. Superscript letters indicate statistically significant differences ($p \leq 0.05$) between the direct extracts and saponified extracts for the same carotenoid compound.

Number correspond with the HPLC-DAD chromatogram peaks (Table 2).

Internal standard.
